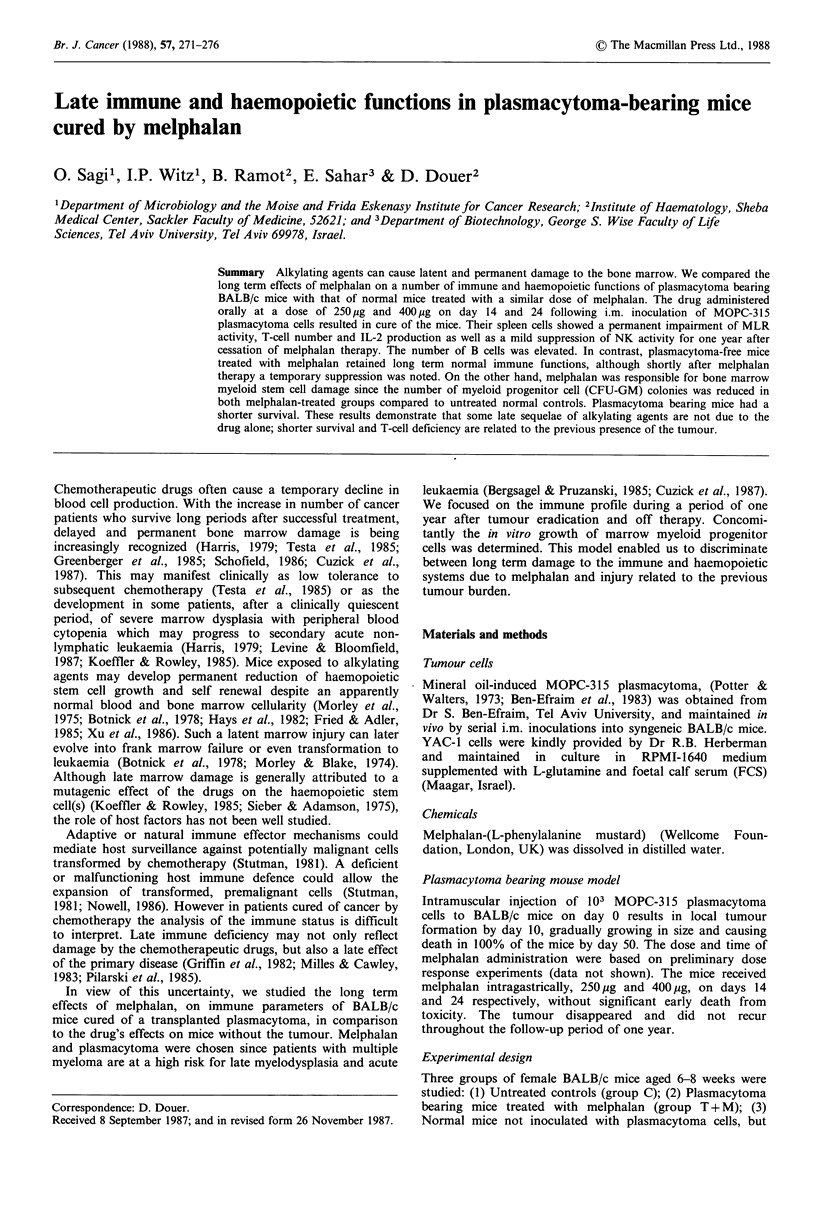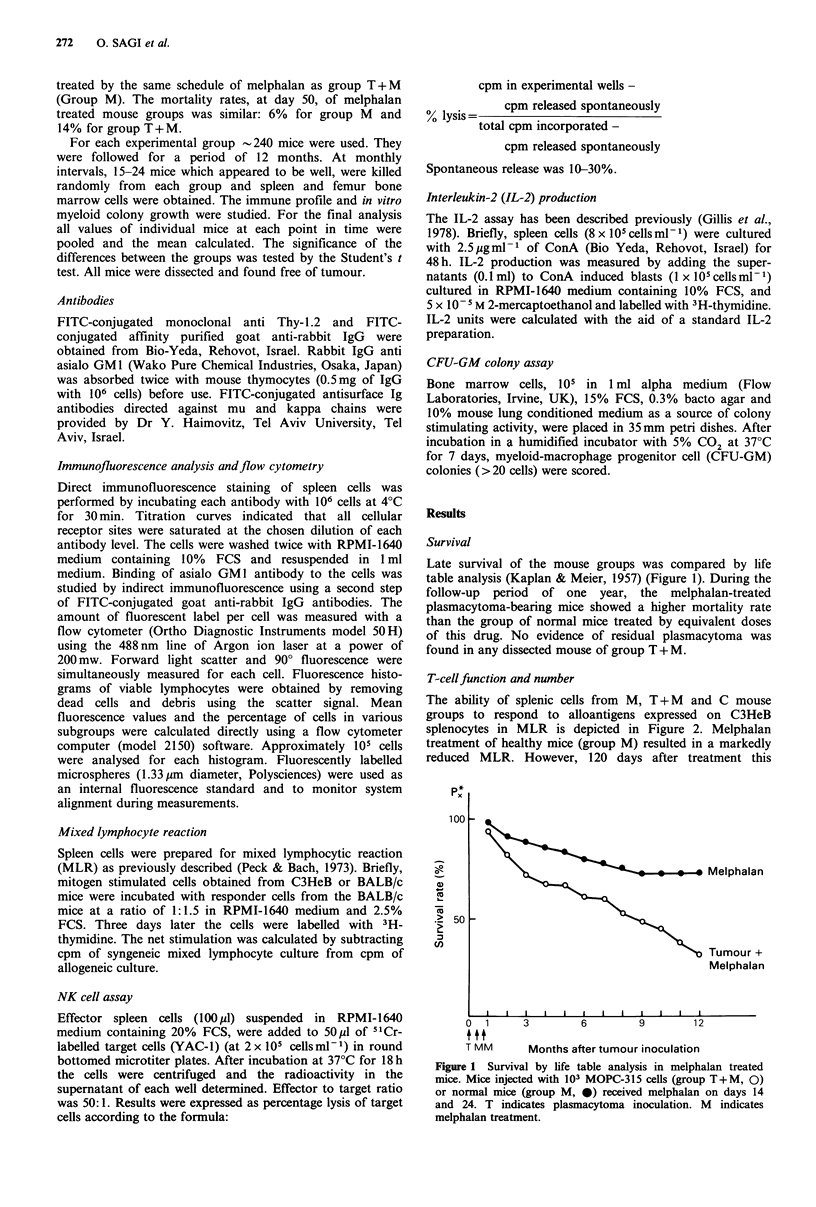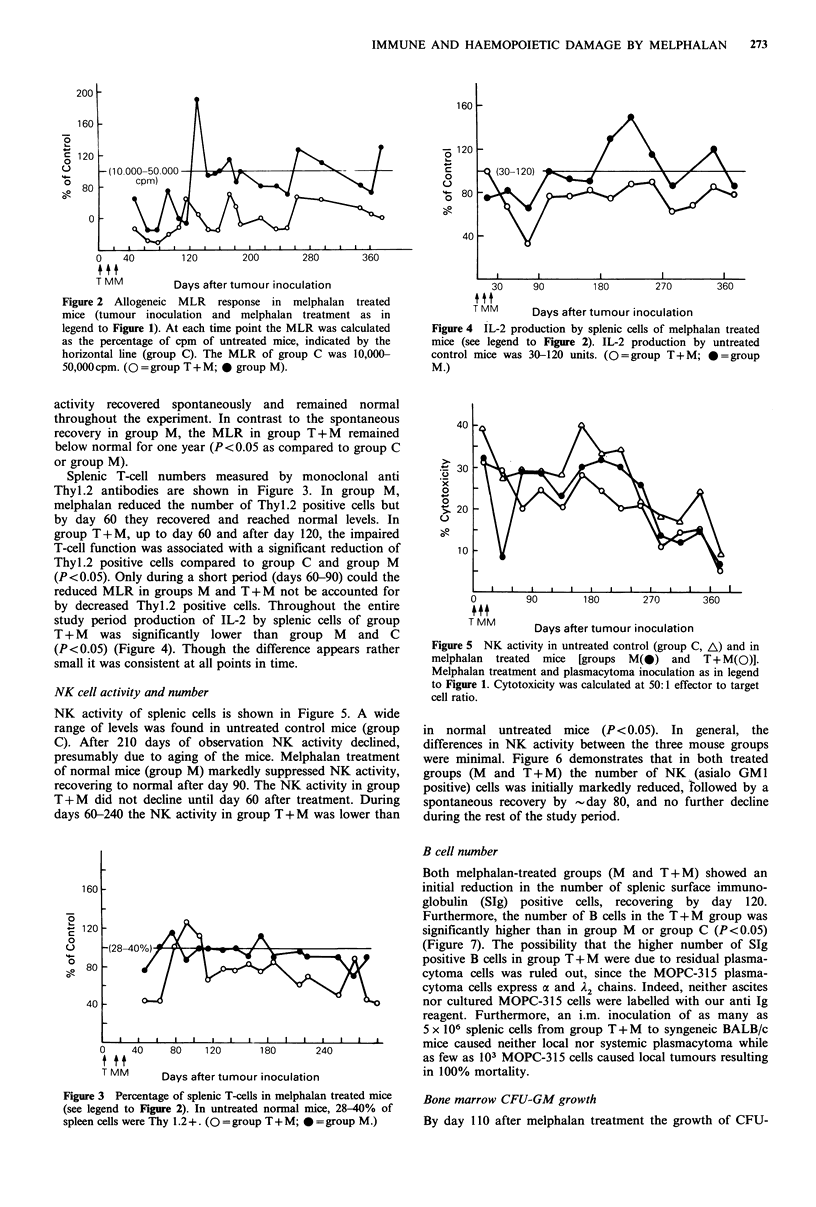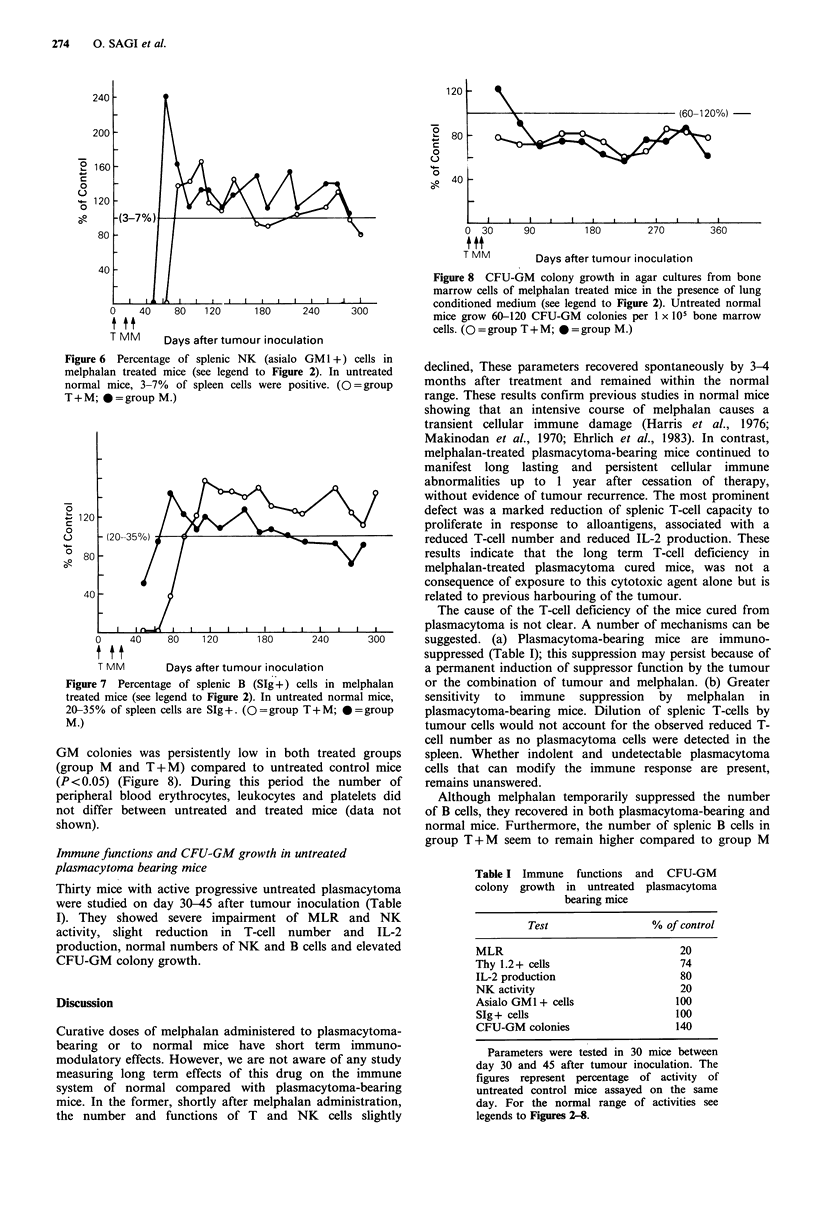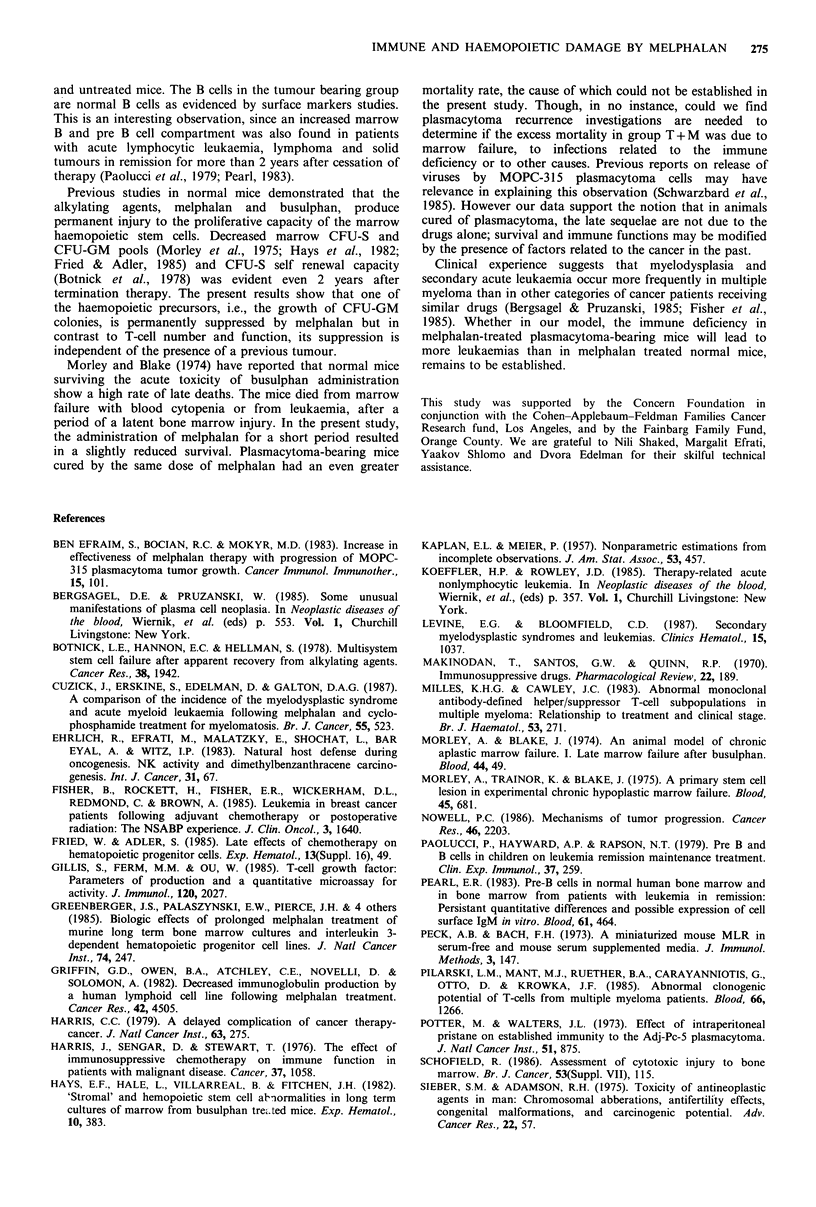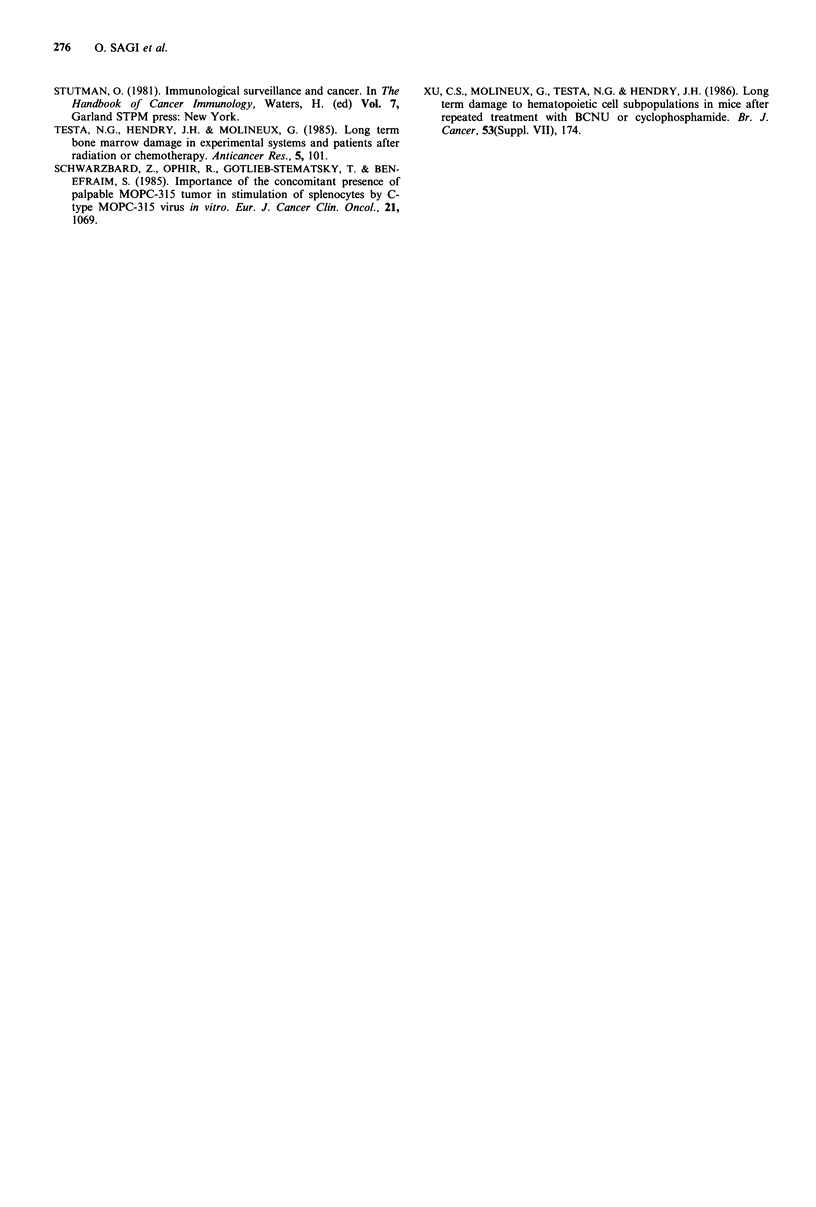# Late immune and haemopoietic functions in plasmacytoma-bearing mice cured by melphalan.

**DOI:** 10.1038/bjc.1988.58

**Published:** 1988-03

**Authors:** O. Sagi, I. P. Witz, B. Ramot, E. Sahar, D. Douer

**Affiliations:** Department of Microbiology, Sheba Medical Center, Sackler Faculty of Medicine, Israel.

## Abstract

Alkylating agents can cause latent and permanent damage to the bone marrow. We compared the long term effects of melphalan on a number of immune and haemopoietic functions of plasmacytoma bearing BALB/c mice with that of normal mice treated with a similar dose of melphalan. The drug administered orally at a dose of 250 micrograms and 400 micrograms on day 14 and 24 following i.m. inoculation of MOPC-315 plasmacytoma cells resulted in cure of the mice. Their spleen cells showed a permanent impairment of MLR activity, T-cell number and IL-2 production as well as a mild suppression of NK activity for one year after cessation of melphalan therapy. The number of B cells was elevated. In contrast, plasmacytoma-free mice treated with melphalan retained long term normal immune functions, although shortly after melphalan therapy a temporary suppression was noted. On the other hand, melphalan was responsible for bone marrow myeloid stem cell damage since the number of myeloid progenitor cell (CFU-GM) colonies was reduced in both melphalan-treated groups compared to untreated normal controls. Plasmacytoma bearing mice had a shorter survival. These results demonstrate that some late sequelae of alkylating agents are not due to the drug alone; shorter survival and T-cell deficiency are related to the previous presence of the tumour.


*Br. J. Cancer* (1988), **57**, 271–276 © The Macmillan Press Ltd., 1988

### Late immune and haemopoietic functions in plasmacytoma-bearing mice cured by melphalan

O. Sagi[1], I.P. Witz[1], B. Ramot[2], E. Sahar[3] & D. Douer[2]

[1]*Department of Microbiology and the Moise and Frida Eskenasy Institute for Cancer Research;* [2]*Institute of Haematology, Sheba Medical Center, Sackler Faculty of Medicine, 52621; and* [3]*Department of Biotechnology, George S. Wise Faculty of Life Sciences, Tel Aviv University, Tel Aviv 69978, Israel.*

**Summary** Alkylating agents can cause latent and permanent damage to the bone marrow. We compared the long term effects of melphalan on a number of immune and haemopoietic functions of plasmacytoma bearing BALB/c mice with that of normal mice treated with a similar dose of melphalan. The drug administered orally at a dose of 250 μg and 400 μg on day 14 and 24 following i.m. inoculation of MOPC-315 plasmacytoma cells resulted in cure of the mice. Their spleen cells showed a permanent impairment of MLR activity, T-cell number and IL-2 production as well as a mild suppression of NK activity for one year after cessation of melphalan therapy. The number of B cells was elevated. In contrast, plasmacytoma-free mice treated with melphalan retained long term normal immune functions, although shortly after melphalan therapy a temporary suppression was noted. On the other hand, melphalan was responsible for bone marrow myeloid stem cell damage since the number of myeloid progenitor cell (CFU-GM) colonies was reduced in both melphalan-treated groups compared to untreated normal controls. Plasmacytoma bearing mice had a shorter survival. These results demonstrate that some late sequelae of alkylating agents are not due to the drug alone; shorter survival and T-cell deficiency are related to the previous presence of the tumour.

Chemotherapeutic drugs often cause a temporary decline in blood cell production. With the increase in number of cancer patients who survive long periods after successful treatment, delayed and permanent bone marrow damage is being increasingly recognized (Harris, 1979; Testa *et al.*, 1985; Greenberger *et al.*, 1985; Schofield, 1986; Cuzick *et al.*, 1987). This may manifest clinically as low tolerance to subsequent chemotherapy (Testa *et al.*, 1985) or as the development in some patients, after a clinically quiescent period, of severe marrow dysplasia with peripheral blood cytopenia which may progress to secondary acute non-lymphatic leukaemia (Harris, 1979; Levine & Bloomfield, 1987; Koeffler & Rowley, 1985). Mice exposed to alkylating agents may develop permanent reduction of haemopoietic stem cell growth and self renewal despite an apparently normal blood and bone marrow cellularity (Morley *et al.*, 1975; Botnick *et al.*, 1978; Hays *et al.*, 1982; Fried & Adler, 1985; Xu *et al.*, 1986). Such a latent marrow injury can later evolve into frank marrow failure or even transformation to leukaemia (Botnick *et al.*, 1978; Morley & Blake, 1974). Although late marrow damage is generally attributed to a mutagenic effect of the drugs on the haemopoietic stem cell(s) (Koeffler & Rowley, 1985; Sieber & Adamson, 1975), the role of host factors has not been well studied.

Adaptive or natural immune effector mechanisms could mediate host surveillance against potentially malignant cells transformed by chemotherapy (Stutman, 1981). A deficient or malfunctioning host immune defence could allow the expansion of transformed, premalignant cells (Stutman, 1981; Nowell, 1986). However in patients cured of cancer by chemotherapy the analysis of the immune status is difficult to interpret. Late immune deficiency may not only reflect damage by the chemotherapeutic drugs, but also a late effect of the primary disease (Griffin *et al.*, 1982; Milles & Cawley, 1983; Pilarski *et al.*, 1985).

In view of this uncertainty, we studied the long term effects of melphalan, on immune parameters of BALB/c mice cured of a transplanted plasmacytoma, in comparison to the drug's effects on mice without the tumour. Melphalan and plasmacytoma were chosen since patients with multiple myeloma are at a high risk for late myelodysplasia and acute leukaemia (Bergsagel & Pruzanski, 1985; Cuzick *et al.*, 1987). We focused on the immune profile during a period of one year after tumour eradication and off therapy. Concomitantly the *in vitro* growth of marrow myeloid progenitor cells was determined. This model enabled us to discriminate between long term damage to the immune and haemopoietic systems due to melphalan and injury related to the previous tumour burden.

Correspondence: D. Douer.
Received 8 September 1987; and in revised form 26 November 1987.

### Materials and methods

### Tumour cells

Mineral oil-induced MOPC-315 plasmacytoma, (Potter & Walters, 1973; Ben-Efraim *et al.*, 1983) was obtained from Dr S. Ben-Efraim, Tel Aviv University, and maintained *in vivo* by serial i.m. inoculations into syngeneic BALB/c mice. YAC-1 cells were kindly provided by Dr R.B. Herberman and maintained in culture in RPMI-1640 medium supplemented with L-glutamine and foetal calf serum (FCS) (Maagar, Israel).

### Chemicals

Melphalan-(L-phenylalanine mustard) (Wellcome Foundation, London, UK) was dissolved in distilled water.

### Plasmacytoma bearing mouse model

Intramuscular injection of $10^3$ MOPC-315 plasmacytoma cells to BALB/c mice on day 0 results in local tumour formation by day 10, gradually growing in size and causing death in 100% of the mice by day 50. The dose and time of melphalan administration were based on preliminary dose response experiments (data not shown). The mice received melphalan intragastrically, 250 μg and 400 μg, on days 14 and 24 respectively, without significant early death from toxicity. The tumour disappeared and did not recur throughout the follow-up period of one year.

### Experimental design

Three groups of female BALB/c mice aged 6–8 weeks were studied: (1) Untreated controls (group C); (2) Plasmacytoma bearing mice treated with melphalan (group T+M); (3) Normal mice not inoculated with plasmacytoma cells, but treated by the same schedule of melphalan as group T+M (Group M). The mortality rates, at day 50, of melphalan treated mouse groups was similar: 6% for group M and 14% for group T+M.

For each experimental group ~240 mice were used. They were followed for a period of 12 months. At monthly intervals, 15–24 mice which appeared to be well, were killed randomly from each group and spleen and femur bone marrow cells were obtained. The immune profile and *in vitro* myeloid colony growth were studied. For the final analysis all values of individual mice at each point in time were pooled and the mean calculated. The significance of the differences between the groups was tested by the Student's *t* test. All mice were dissected and found free of tumour.

*Antibodies*

FITC-conjugated monoclonal anti Thy-1.2 and FITC-conjugated affinity purified goat anti-rabbit IgG were obtained from Bio-Yeda, Rehovot, Israel. Rabbit IgG anti asialo GM1 (Wako Pure Chemical Industries, Osaka, Japan) was absorbed twice with mouse thymocytes (0.5 mg of IgG with $10^6$ cells) before use. FITC-conjugated antisurface Ig antibodies directed against mu and kappa chains were provided by Dr Y. Haimovitz, Tel Aviv University, Tel Aviv, Israel.

*Immunofluorescence analysis and flow cytometry*

Direct immunofluorescence staining of spleen cells was performed by incubating each antibody with $10^6$ cells at 4°C for 30 min. Titration curves indicated that all cellular receptor sites were saturated at the chosen dilution of each antibody level. The cells were washed twice with RPMI-1640 medium containing 10% FCS and resuspended in 1 ml medium. Binding of asialo GM1 antibody to the cells was studied by indirect immunofluorescence using a second step of FITC-conjugated goat anti-rabbit IgG antibodies. The amount of fluorescent label per cell was measured with a flow cytometer (Ortho Diagnostic Instruments model 50 H) using the 488 nm line of Argon ion laser at a power of 200 mw. Forward light scatter and 90° fluorescence were simultaneously measured for each cell. Fluorescence histograms of viable lymphocytes were obtained by removing dead cells and debris using the scatter signal. Mean fluorescence values and the percentage of cells in various subgroups were calculated directly using a flow cytometer computer (model 2150) software. Approximately $10^5$ cells were analysed for each histogram. Fluorescently labelled microspheres (1.33 $\mu$m diameter, Polysciences) were used as an internal fluorescence standard and to monitor system alignment during measurements.

*Mixed lymphocyte reaction*

Spleen cells were prepared for mixed lymphocytic reaction (MLR) as previously described (Peck & Bach, 1973). Briefly, mitogen stimulated cells obtained from C3HeB or BALB/c mice were incubated with responder cells from the BALB/c mice at a ratio of 1:1.5 in RPMI-1640 medium and 2.5% FCS. Three days later the cells were labelled with $^3$H-thymidine. The net stimulation was calculated by subtracting cpm of syngeneic mixed lymphocyte culture from cpm of allogeneic culture.

*NK cell assay*

Effector spleen cells (100 $\mu$l) suspended in RPMI-1640 medium containing 20% FCS, were added to 50 $\mu$l of $^{51}$Cr-labelled target cells (YAC-1) (at $2 \times 10^5$ cells ml$^{-1}$) in round bottomed microtiter plates. After incubation at 37°C for 18 h the cells were centrifuged and the radioactivity in the supernatant of each well determined. Effector to target ratio was 50:1. Results were expressed as percentage lysis of target cells according to the formula:

$$\%\ \text{lysis} = \frac{\text{cpm in experimental wells} - \text{cpm released spontaneously}}{\text{total cpm incorporated} - \text{cpm released spontaneously}}$$

Spontaneous release was 10–30%.

*Interleukin-2 (IL-2) production*

The IL-2 assay has been described previously (Gillis *et al.*, 1978). Briefly, spleen cells ($8 \times 10^5$ cells ml$^{-1}$) were cultured with 2.5 $\mu$g ml$^{-1}$ of ConA (Bio Yeda, Rehovot, Israel) for 48 h. IL-2 production was measured by adding the supernatants (0.1 ml) to ConA induced blasts ($1 \times 10^5$ cells ml$^{-1}$) cultured in RPMI-1640 medium containing 10% FCS, and $5 \times 10^{-5}$ M 2-mercaptoethanol and labelled with $^3$H-thymidine. IL-2 units were calculated with the aid of a standard IL-2 preparation.

*CFU-GM colony assay*

Bone marrow cells, $10^5$ in 1 ml alpha medium (Flow Laboratories, Irvine, UK), 15% FCS, 0.3% bacto agar and 10% mouse lung conditioned medium as a source of colony stimulating activity, were placed in 35 mm petri dishes. After incubation in a humidified incubator with 5% $CO_2$ at 37°C for 7 days, myeloid-macrophage progenitor cell (CFU-GM) colonies (>20 cells) were scored.

### Results

*Survival*

Late survival of the mouse groups was compared by life table analysis (Kaplan & Meier, 1957) (Figure 1). During the follow-up period of one year, the melphalan-treated plasmacytoma-bearing mice showed a higher mortality rate than the group of normal mice treated by equivalent doses of this drug. No evidence of residual plasmacytoma was found in any dissected mouse of group T+M.

*T-cell function and number*

The ability of splenic cells from M, T+M and C mouse groups to respond to alloantigens expressed on C3HeB splenocytes in MLR is depicted in Figure 2. Melphalan treatment of healthy mice (group M) resulted in a markedly reduced MLR. However, 120 days after treatment this

**Figure 1** Survival by life table analysis in melphalan treated mice. Mice injected with $10^3$ MOPC-315 cells (group T+M, ○) or normal mice (group M, ●) received melphalan on days 14 and 24. T indicates plasmacytoma inoculation. M indicates melphalan treatment.

**Figure 2** Allogeneic MLR response in melphalan treated mice (tumour inoculation and melphalan treatment as in legend to **Figure 1**). At each time point the MLR was calculated as the percentage of cpm of untreated mice, indicated by the horizontal line (group C). The MLR of group C was 10,000–50,000 cpm. (○ = group T + M; ● group M).

**Figure 4** IL-2 production by splenic cells of melphalan treated mice (see legend to **Figure 2**). IL-2 production by untreated control mice was 30–120 units. (○ = group T + M; ● = group M.)

activity recovered spontaneously and remained normal throughout the experiment. In contrast to the spontaneous recovery in group M, the MLR in group T + M remained below normal for one year ($P < 0.05$ as compared to group C or group M).

Splenic T-cell numbers measured by monoclonal anti Thy1.2 antibodies are shown in Figure 3. In group M, melphalan reduced the number of Thy1.2 positive cells but by day 60 they recovered and reached normal levels. In group T + M, up to day 60 and after day 120, the impaired T-cell function was associated with a significant reduction of Thy1.2 positive cells compared to group C and group M ($P < 0.05$). Only during a short period (days 60–90) could the reduced MLR in groups M and T + M not be accounted for by decreased Thy1.2 positive cells. Throughout the entire study period production of IL-2 by splenic cells of group T + M was significantly lower than group M and C ($P < 0.05$) (Figure 4). Though the difference appears rather small it was consistent at all points in time.

*NK cell activity and number*

NK activity of splenic cells is shown in Figure 5. A wide range of levels was found in untreated control mice (group C). After 210 days of observation NK activity declined, presumably due to aging of the mice. Melphalan treatment of normal mice (group M) markedly suppressed NK activity, recovering to normal after day 90. The NK activity in group T + M did not decline until day 60 after treatment. During days 60–240 the NK activity in group T + M was lower than

**Figure 5** NK activity in untreated control (group C, △) and in melphalan treated mice [groups M(●) and T + M(○)]. Melphalan treatment and plasmacytoma inoculation as in legend to **Figure 1**. Cytotoxicity was calculated at 50:1 effector to target cell ratio.

in normal untreated mice ($P < 0.05$). In general, the differences in NK activity between the three mouse groups were minimal. Figure 6 demonstrates that in both treated groups (M and T + M) the number of NK (asialo GM1 positive) cells was initially markedly reduced, followed by a spontaneous recovery by ~day 80, and no further decline during the rest of the study period.

**Figure 3** Percentage of splenic T-cells in melphalan treated mice (see legend to **Figure 2**). In untreated normal mice, 28–40% of spleen cells were Thy 1.2+. (○ = group T + M; ● = group M.)

*B cell number*

Both melphalan-treated groups (M and T + M) showed an initial reduction in the number of splenic surface immunoglobulin (SIg) positive cells, recovering by day 120. Furthermore, the number of B cells in the T + M group was significantly higher than in group M or group C ($P < 0.05$) (Figure 7). The possibility that the higher number of SIg positive B cells in group T + M were due to residual plasmacytoma cells was ruled out, since the MOPC-315 plasmacytoma cells express $\alpha$ and $\lambda_2$ chains. Indeed, neither ascites nor cultured MOPC-315 cells were labelled with our anti Ig reagent. Furthermore, an i.m. inoculation of as many as $5 \times 10^6$ splenic cells from group T + M to syngeneic BALB/c mice caused neither local nor systemic plasmacytoma while as few as $10^3$ MOPC-315 cells caused local tumours resulting in 100% mortality.

*Bone marrow CFU-GM growth*

By day 110 after melphalan treatment the growth of CFU-

**Figure 6** Percentage of splenic NK (asialo GM1+) cells in melphalan treated mice (see legend to **Figure 2**). In untreated normal mice, 3–7% of spleen cells were positive. (○ = group T+M; ● = group M.)

**Figure 7** Percentage of splenic B (SIg+) cells in melphalan treated mice (see legend to **Figure 2**). In untreated normal mice, 20–35% of spleen cells are SIg+. (○ = group T+M; ● = group M.)

GM colonies was persistently low in both treated groups (group M and T+M) compared to untreated control mice ($P<0.05$) (Figure 8). During this period the number of peripheral blood erythrocytes, leukocytes and platelets did not differ between untreated and treated mice (data not shown).

*Immune functions and CFU-GM growth in untreated plasmacytoma bearing mice*

Thirty mice with active progressive untreated plasmacytoma were studied on day 30–45 after tumour inoculation (Table I). They showed severe impairment of MLR and NK activity, slight reduction in T-cell number and IL-2 production, normal numbers of NK and B cells and elevated CFU-GM colony growth.

### Discussion

Curative doses of melphalan administered to plasmacytoma-bearing or to normal mice have short term immunomodulatory effects. However, we are not aware of any study measuring long term effects of this drug on the immune system of normal compared with plasmacytoma-bearing mice. In the former, shortly after melphalan administration, the number and functions of T and NK cells slightly

**Figure 8** CFU-GM colony growth in agar cultures from bone marrow cells of melphalan treated mice in the presence of lung conditioned medium (see legend to **Figure 2**). Untreated normal mice grow 60–120 CFU-GM colonies per $1\times10^5$ bone marrow cells. (○ = group T+M; ● = group M.)

declined, These parameters recovered spontaneously by 3–4 months after treatment and remained within the normal range. These results confirm previous studies in normal mice showing that an intensive course of melphalan causes a transient cellular immune damage (Harris *et al.*, 1976; Makinodan *et al.*, 1970; Ehrlich *et al.*, 1983). In contrast, melphalan-treated plasmacytoma-bearing mice continued to manifest long lasting and persistent cellular immune abnormalities up to 1 year after cessation of therapy, without evidence of tumour recurrence. The most prominent defect was a marked reduction of splenic T-cell capacity to proliferate in response to alloantigens, associated with a reduced T-cell number and reduced IL-2 production. These results indicate that the long term T-cell deficiency in melphalan-treated plasmacytoma cured mice, was not a consequence of exposure to this cytotoxic agent alone but is related to previous harbouring of the tumour.

The cause of the T-cell deficiency of the mice cured from plasmacytoma is not clear. A number of mechanisms can be suggested. (a) Plasmacytoma-bearing mice are immunosuppressed (Table I); this suppression may persist because of a permanent induction of suppressor function by the tumour or the combination of tumour and melphalan. (b) Greater sensitivity to immune suppression by melphalan in plasmacytoma-bearing mice. Dilution of splenic T-cells by tumour cells would not account for the observed reduced T-cell number as no plasmacytoma cells were detected in the spleen. Whether indolent and undetectable plasmacytoma cells that can modify the immune response are present, remains unanswered.

Although melphalan temporarily suppressed the number of B cells, they recovered in both plasmacytoma-bearing and normal mice. Furthermore, the number of splenic B cells in group T+M seem to remain higher compared to group M

**Table I** Immune functions and CFU-GM colony growth in untreated plasmacytoma bearing mice

| *Test* | *% of control* |
|---|---|
| MLR | 20 |
| Thy 1.2+ cells | 74 |
| IL-2 production | 80 |
| NK activity | 20 |
| Asialo GM1+ cells | 100 |
| SIg+ cells | 100 |
| CFU-GM colonies | 140 |

Parameters were tested in 30 mice between day 30 and 45 after tumour inoculation. The figures represent percentage of activity of untreated control mice assayed on the same day. For the normal range of activities see legends to **Figures 2–8**.

and untreated mice. The B cells in the tumour bearing group are normal B cells as evidenced by surface markers studies. This is an interesting observation, since an increased marrow B and pre B cell compartment was also found in patients with acute lymphocytic leukaemia, lymphoma and solid tumours in remission for more than 2 years after cessation of therapy (Paolucci *et al.*, 1979; Pearl, 1983).

Previous studies in normal mice demonstrated that the alkylating agents, melphalan and busulphan, produce permanent injury to the proliferative capacity of the marrow haemopoietic stem cells. Decreased marrow CFU-S and CFU-GM pools (Morley *et al.*, 1975; Hays *et al.*, 1982; Fried & Adler, 1985) and CFU-S self renewal capacity (Botnick *et al.*, 1978) was evident even 2 years after termination therapy. The present results show that one of the haemopoietic precursors, i.e., the growth of CFU-GM colonies, is permanently suppressed by melphalan but in contrast to T-cell number and function, its suppression is independent of the presence of a previous tumour.

Morley and Blake (1974) have reported that normal mice surviving the acute toxicity of busulphan administration show a high rate of late deaths. The mice died from marrow failure with blood cytopenia or from leukaemia, after a period of a latent bone marrow injury. In the present study, the administration of melphalan for a short period resulted in a slightly reduced survival. Plasmacytoma-bearing mice cured by the same dose of melphalan had an even greater mortality rate, the cause of which could not be established in the present study. Though, in no instance, could we find plasmacytoma recurrence investigations are needed to determine if the excess mortality in group T+M was due to marrow failure, to infections related to the immune deficiency or to other causes. Previous reports on release of viruses by MOPC-315 plasmacytoma cells may have relevance in explaining this observation (Schwarzbard *et al.*, 1985). However our data support the notion that in animals cured of plasmacytoma, the late sequelae are not due to the drugs alone; survival and immune functions may be modified by the presence of factors related to the cancer in the past.

Clinical experience suggests that myelodysplasia and secondary acute leukaemia occur more frequently in multiple myeloma than in other categories of cancer patients receiving similar drugs (Bergsagel & Pruzanski, 1985; Fisher *et al.*, 1985). Whether in our model, the immune deficiency in melphalan-treated plasmacytoma-bearing mice will lead to more leukaemias than in melphalan treated normal mice, remains to be established.

This study was supported by the Concern Foundation in conjunction with the Cohen–Applebaum–Feldman Families Cancer Research fund, Los Angeles, and by the Fainbarg Family Fund, Orange County. We are grateful to Nili Shaked, Margalit Efrati, Yaakov Shlomo and Dvora Edelman for their skilful technical assistance.

### References

BEN EFRAIM, S., BOCIAN, R.C. & MOKYR, M.D. (1983). Increase in effectiveness of melphalan therapy with progression of MOPC-315 plasmacytoma tumor growth. *Cancer Immunol. Immunother.*, **15**, 101.

BERGSAGEL, D.E. & PRUZANSKI, W. (1985). Some unusual manifestations of plasma cell neoplasia. In *Neoplastic diseases of the blood*, Wiernik, *et al.* (eds) p. 553. **Vol. 1**, Churchill Livingstone: New York.

BOTNICK, L.E., HANNON, E.C. & HELLMAN, S. (1978). Multisystem stem cell failure after apparent recovery from alkylating agents. *Cancer Res.*, **38**, 1942.

CUZICK, J., ERSKINE, S., EDELMAN, D. & GALTON, D.A.G. (1987). A comparison of the incidence of the myelodysplastic syndrome and acute myeloid leukaemia following melphalan and cyclophosphamide treatment for myelomatosis. *Br. J. Cancer*, **55**, 523.

EHRLICH, R., EFRATI, M., MALATZKY, E., SHOCHAT, L., BAR EYAL, A. & WITZ, I.P. (1983). Natural host defense during oncogenesis. NK activity and dimethylbenzanthracene carcinogenesis. *Int. J. Cancer*, **31**, 67.

FISHER, B., ROCKETT, H., FISHER, E.R., WICKERHAM, D.L., REDMOND, C. & BROWN, A. (1985). Leukemia in breast cancer patients following adjuvant chemotherapy or postoperative radiation: The NSABP experience. *J. Clin. Oncol.*, **3**, 1640.

FRIED, W. & ADLER, S. (1985). Late effects of chemotherapy on hematopoietic progenitor cells. *Exp. Hematol.*, **13**(Suppl. 16), 49.

GILLIS, S., FERM, M.M. & OU, W. (1985). T-cell growth factor: Parameters of production and a quantitative microassay for activity. *J. Immunol.*, **120**, 2027.

GREENBERGER, J.S., PALASZYNSKI, E.W., PIERCE, J.H. & 4 others (1985). Biologic effects of prolonged melphalan treatment of murine long term bone marrow cultures and interleukin 3-dependent hematopoietic progenitor cell lines. *J. Natl Cancer Inst.*, **74**, 247.

GRIFFIN, G.D., OWEN, B.A., ATCHLEY, C.E., NOVELLI, D. & SOLOMON, A. (1982). Decreased immunoglobulin production by a human lymphoid cell line following melphalan treatment. *Cancer Res.*, **42**, 4505.

HARRIS, C.C. (1979). A delayed complication of cancer therapy-cancer. *J. Natl Cancer Inst.*, **63**, 275.

HARRIS, J., SENGAR, D. & STEWART, T. (1976). The effect of immunosuppressive chemotherapy on immune function in patients with malignant disease. *Cancer*, **37**, 1058.

HAYS, E.F., HALE, L., VILLARREAL, B. & FITCHEN, J.H. (1982). 'Stromal' and hemopoietic stem cell abnormalities in long term cultures of marrow from busulphan treated mice. *Exp. Hematol.*, **10**, 383.

KAPLAN, E.L. & MEIER, P. (1957). Nonparametric estimations from incomplete observations. *J. Am. Stat. Assoc.*, **53**, 457.

KOEFFLER, H.P. & ROWLEY, J.D. (1985). Therapy-related acute nonlymphocytic leukemia. In *Neoplastic diseases of the blood*, Wiernik, *et al.*, (eds) p. 357. **Vol. 1**, Churchill Livingstone: New York.

LEVINE, E.G. & BLOOMFIELD, C.D. (1987). Secondary myelodysplastic syndromes and leukemias. *Clinics Hematol.*, **15**, 1037.

MAKINODAN, T., SANTOS, G.W. & QUINN, R.P. (1970). Immunosuppressive drugs. *Pharmacological Review*, **22**, 189.

MILLES, K.H.G. & CAWLEY, J.C. (1983). Abnormal monoclonal antibody-defined helper/suppressor T-cell subpopulations in multiple myeloma: Relationship to treatment and clinical stage. *Br. J. Haematol.*, **53**, 271.

MORLEY, A. & BLAKE, J. (1974). An animal model of chronic aplastic marrow failure. I. Late marrow failure after busulphan. *Blood*, **44**, 49.

MORLEY, A., TRAINOR, K. & BLAKE, J. (1975). A primary stem cell lesion in experimental chronic hypoplastic marrow failure. *Blood*, **45**, 681.

NOWELL, P.C. (1986). Mechanisms of tumor progression. *Cancer Res.*, **46**, 2203.

PAOLUCCI, P., HAYWARD, A.P. & RAPSON, N.T. (1979). Pre B and B cells in children on leukemia remission maintenance treatment. *Clin. Exp. Immunol.*, **37**, 259.

PEARL, E.R. (1983). Pre-B cells in normal human bone marrow and in bone marrow from patients with leukemia in remission: Persistant quantitative differences and possible expression of cell surface IgM *in vitro*. *Blood*, **61**, 464.

PECK, A.B. & BACH, F.H. (1973). A miniaturized mouse MLR in serum-free and mouse serum supplemented media. *J. Immunol. Methods*, **3**, 147.

PILARSKI, L.M., MANT, M.J., RUETHER, B.A., CARAYANNIOTIS, G., OTTO, D. & KROWKA, J.F. (1985). Abnormal clonogenic potential of T-cells from multiple myeloma patients. *Blood*, **66**, 1266.

POTTER, M. & WALTERS, J.L. (1973). Effect of intraperitoneal pristane on established immunity to the Adj-Pc-5 plasmacytoma. *J. Natl Cancer Inst.*, **51**, 875.

SCHOFIELD, R. (1986). Assessment of cytotoxic injury to bone marrow. *Br. J. Cancer*, **53**(Suppl. VII), 115.

SIEBER, S.M. & ADAMSON, R.H. (1975). Toxicity of antineoplastic agents in man: Chromosomal abberations, antifertility effects, congenital malformations, and carcinogenic potential. *Adv. Cancer Res.*, **22**, 57.

STUTMAN, O. (1981). Immunological surveillance and cancer. In *The Handbook of Cancer Immunology,* Waters, H. (ed) **Vol. 7,** Garland STPM press: New York.

TESTA, N.G., HENDRY, J.H. & MOLINEUX, G. (1985). Long term bone marrow damage in experimental systems and patients after radiation or chemotherapy. *Anticancer Res.,* **5,** 101.

SCHWARZBARD, Z., OPHIR, R., GOTLIEB-STEMATSKY, T. & BEN-EFRAIM, S. (1985). Importance of the concomitant presence of palpable MOPC-315 tumor in stimulation of splenocytes by C-type MOPC-315 virus *in vitro. Eur. J. Cancer Clin. Oncol.,* **21,** 1069.

XU, C.S., MOLINEUX, G., TESTA, N.G. & HENDRY, J.H. (1986). Long term damage to hematopoietic cell subpopulations in mice after repeated treatment with BCNU or cyclophosphamide. *Br. J. Cancer,* **53**(Suppl. VII), 174.